# Supplementary figures and images for: Coding of object location by heterogeneous neural populations with spatially dependent correlations in weakly electric fish
Source: PLoS Comput Biol. 2023 Mar 3;19(3):e1010938. doi: 10.1371/journal.pcbi.1010938 (PMC10016687; doi:10.1371/journal.pcbi.1010938)

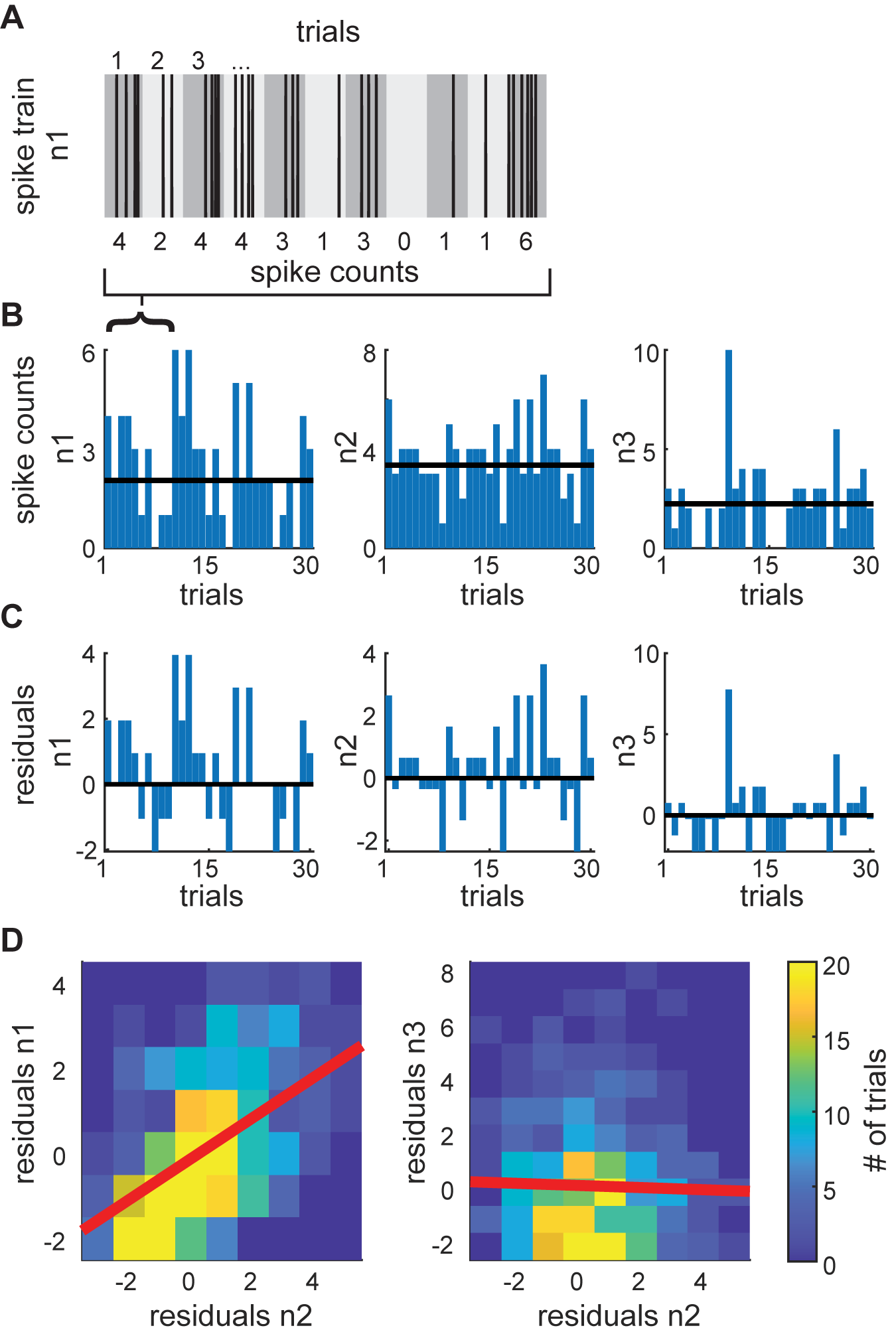

Supplement: S1 Fig — (A) The spike train of an example neuron (n1) is shown, with each trial indicated by alternating grey and white bars and the trial number above. The spike counts for each trial are indicated below. (B) The spike counts, or firing rate, of 3 example neurons (n1 left, n2 middle and n3 right) are shown for 30 trials each. The trial-averaged firing rate over all 200 trials in the data is also shown (black horizontal line) for each neuron. (C) The residual spike counts (the spike counts for each trial minus the trial-averaged firing rate) vary around zero. (D) The color plot shows the residuals of one neuron vs another. The color shows the number of trials in each square. There is a strong positive spike-count correlation for the pair on the left and no significant correlation for the pair on the right (red fitted line: left pair: n1 and n2, rSC = 0.51, p = 1.1 · 10−27; right pair: n2 and n3, rSC = - 0.03, p = 0.54). Showing that for the pair on the left, when one neuron tends to fire above its mean, the other does as well, whereas the pair on the right tend to vary around their respective trial-averaged firing rate independent from each other. (TIF) [file pcbi.1010938.s001.tif]

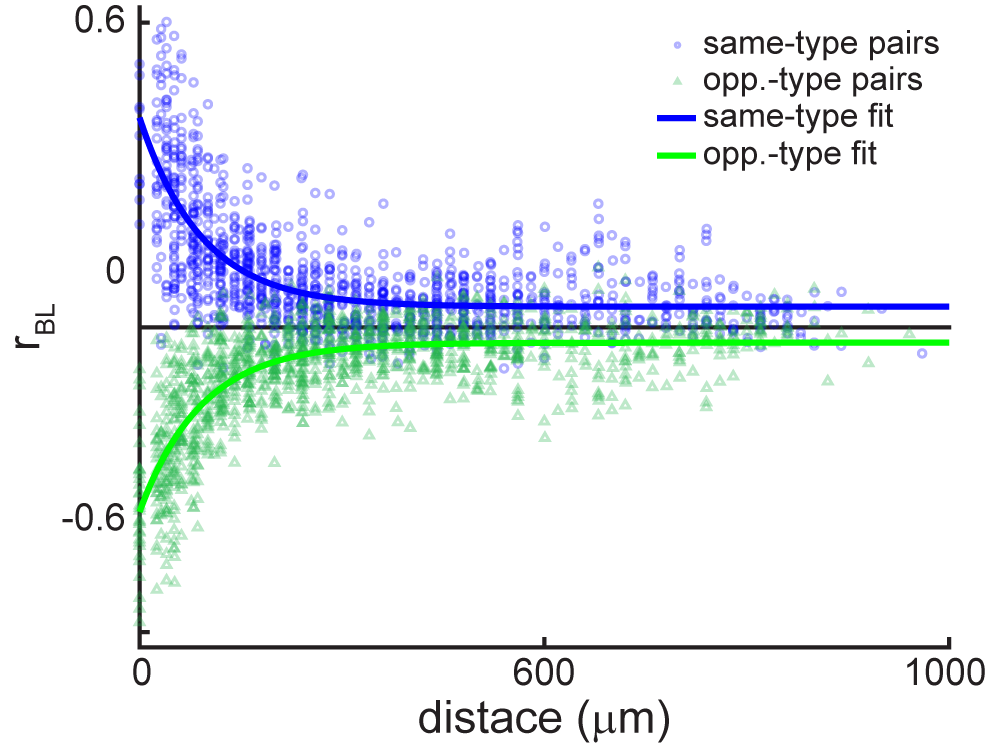

Supplement: S2 Fig — Pairwise baseline correlation (rBL) values approach zero as a function of increasing relative distance between neurons. The baseline correlation values of same-type pairs (ON-ON and OFF-OFF; blue circles) follow an exponentially decreasing trend as a function of distance (blue fitted line) and the opposite-type pairs (ON-OFF; green triangles) show the same exponential trend approaching zero but from the negative direction (green fitted line). In the case of opposite-type pairs it is important to note that the receptive fields are opposite as well because ON and OFF type pairs fire out of phase of each other, therefore the relationship between the correlations and receptive fields are qualitatively similar for all pair types. (TIF) [file pcbi.1010938.s002.tif]

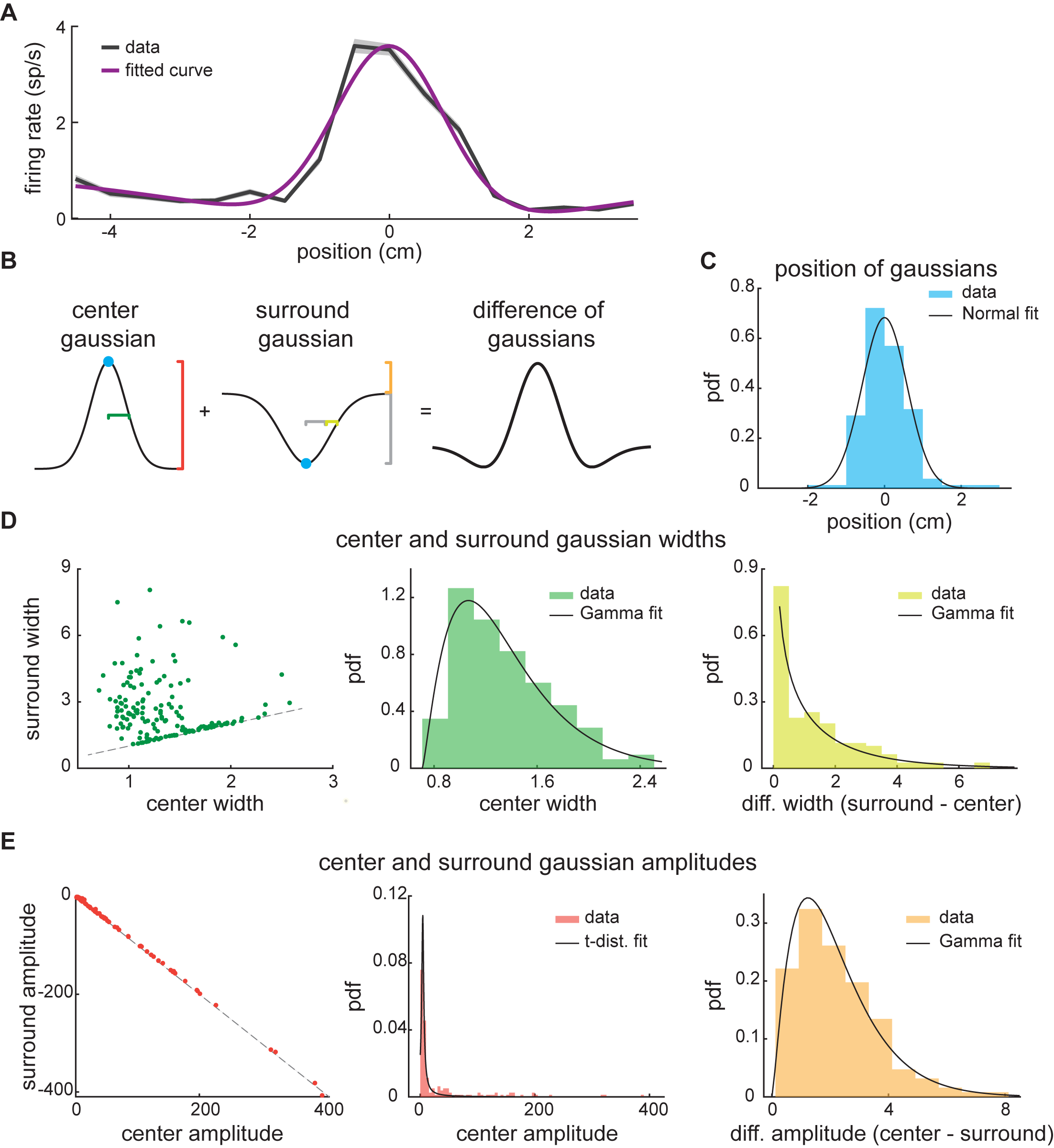

Supplement: S3 Fig — (A) Example ON-type receptive field (black) is shown with the fitted difference of Gaussians curve (purple). (B) Schematic showing the parameters of the difference of Gaussians fit: the left and middle curves (black) show the center and surround Gaussians and the right curve (black) shows the fitted receptive field. The blue circles mark the center position of the receptive field. The dark green horizontal bracket is the width of the center, the light green bracket is the difference between the widths of the center and surround. The vertical dark orange bracket is the center amplitude, and the light orange bracket is the difference between the center and surround amplitude. (C) Receptive field positions: The distribution of the positions of all neurons pooled across sessions (blue) was fitted with a normal distribution (black). (D) Receptive field widths: For all fitted neurons, the width of the surround Gaussian is larger than the width of the center as the data (left panel; green dots) falls above the unity line (dashed grey line). Therefore, the distribution of the center Gaussian widths was fitted with a Gamma distribution as was the distribution of the difference between the two (surround-center). After drawing randomly from both distributions, the surround was modeled as the sum of the two. (E) Receptive field amplitudes: The surround amplitude vs center amplitude (left panel) demonstrates that the center amplitude is always larger than the surround amplitude (orange dots lie above the dashed grey unity line). The distribution of center amplitudes (middle panel; dark orange) was fitted with a t-distribution and the difference distribution (center–surround) was fitted with a Gamma distribution (right panel; light orange). After drawing randomly from both distributions, the first was used as the center amplitude and the second was subtracted from the first to model the surround amplitude. (For all distribution parameters see Materials and Methods.) (TIF) [file pcbi.1010938.s003.tif]

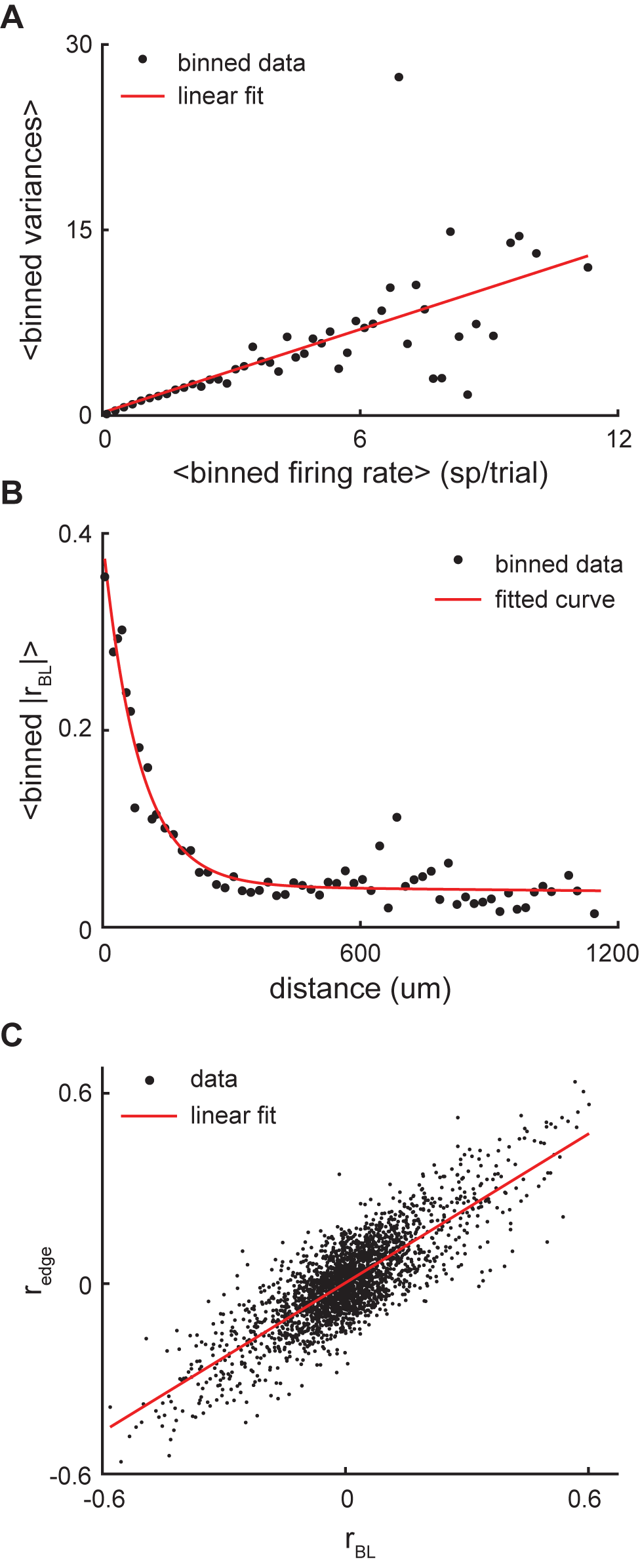

Supplement: S4 Fig — (A) The variances of the neural firing rates in the data increase with the average firing rate. Prior to fitting, the data was binned and averaged (black dots) then fit with a linear function (red line). (B) To fit the |rBL| as a function of relative distance, the data was binned and averaged, and a sum of exponentials was fitted (red curve). (C) To confirm that as the stimulus approaches the edge of the population of receptive fields, the rSC approach rBL, the spike-count correlations at the stimulus positions at the rostral and caudal edges of the recording positions were visualized vs the rBL, which shows a strong linear relationship (see Materials and Methods for details.) (TIF) [file pcbi.1010938.s004.tif]

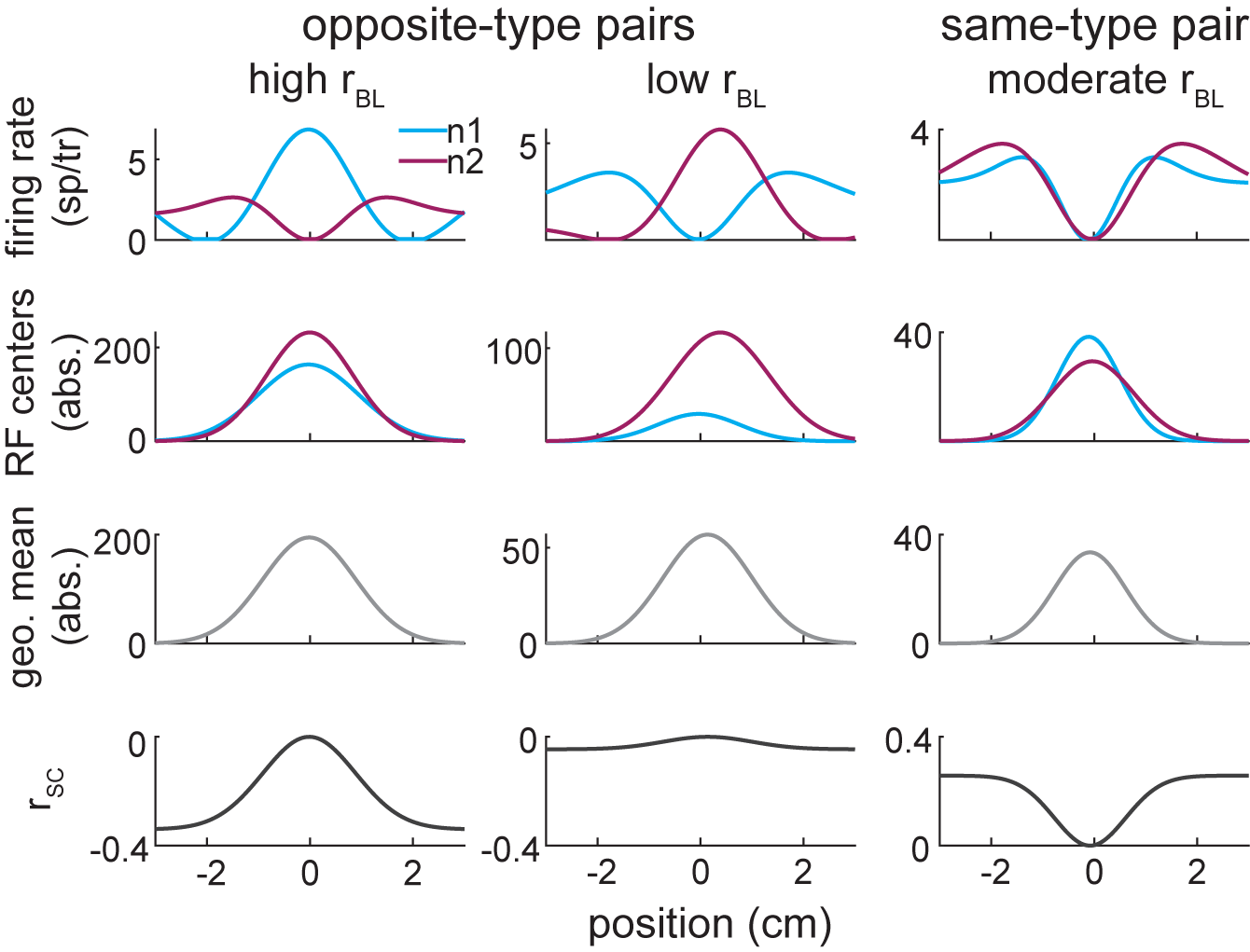

Supplement: S5 Fig — The spatially dependent correlations are modeled as a function of the overlap of the pair of receptive fields (top row; neuron 1 blue, neuron 2 purple). The geometric mean (3rd row; grey) of the absolute value of the receptive field centers (2nd row) is normalized to range between the baseline correlation assigned to that pair and zero (bottom row; black). Three examples are provided: a high rBL, opposite-type pair (left), a low rBL, opposite-type pair (middle), and a moderate rBL, same-type pair (right). (TIF) [file pcbi.1010938.s005.tif]

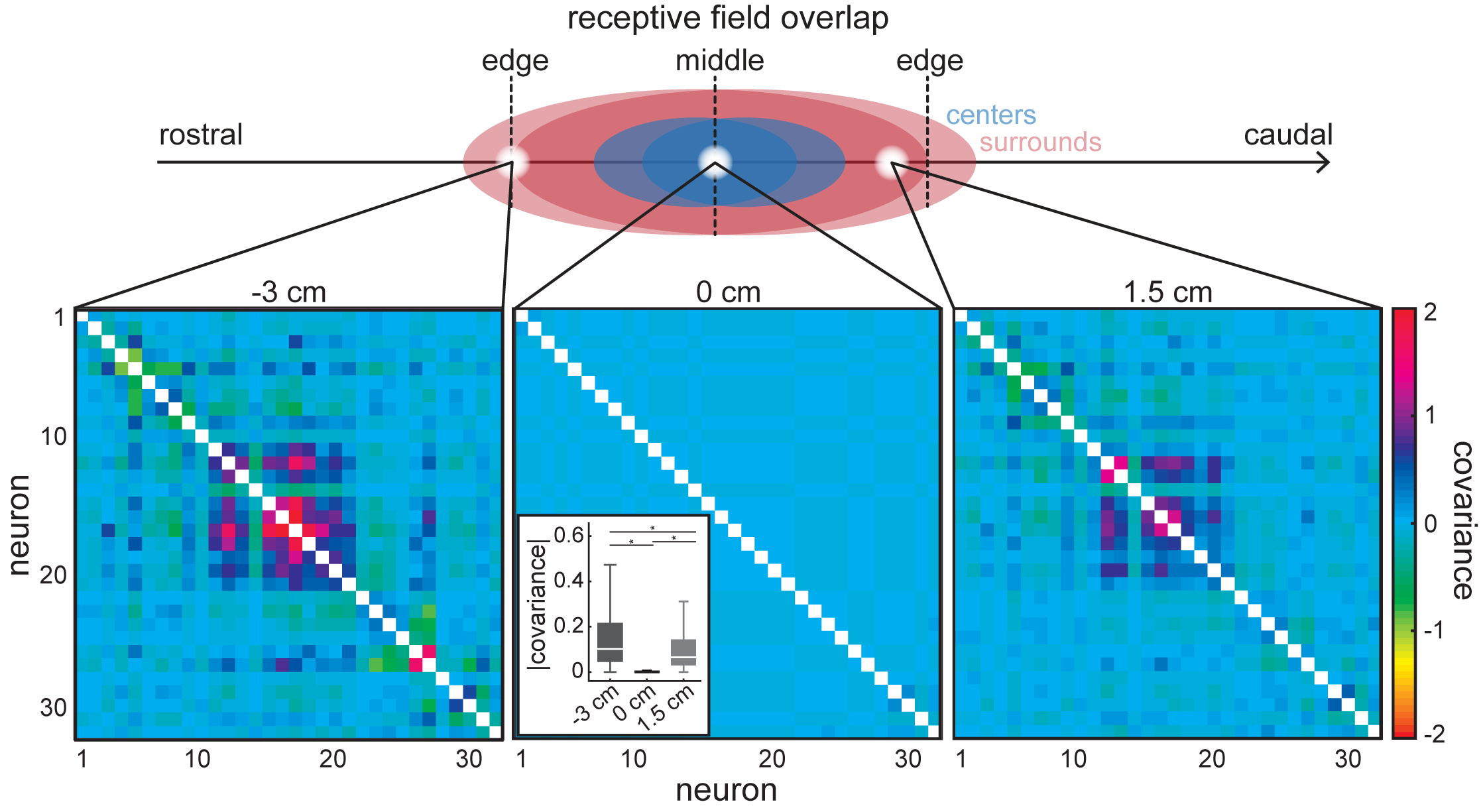

Supplement: S6 Fig — The model covariances are shown for three different stimulus positions: at the center of the population of receptive fields where there is primarily center-center overlap (center panel) and near the edges of the population of receptive fields where there is surround-surround overlap (right panel) or minimal to no overlap (left panel). These results demonstrate that the covariances calculated from the modeled variances and correlations reproduce trends seen in the data. Inset: the distributions of the covariance magnitudes are significantly different across these three stimulus positions (Kruskal Wallis: p = 1.5 · 10−190). “*” indicates statistical significance. (TIF) [file pcbi.1010938.s006.tif]

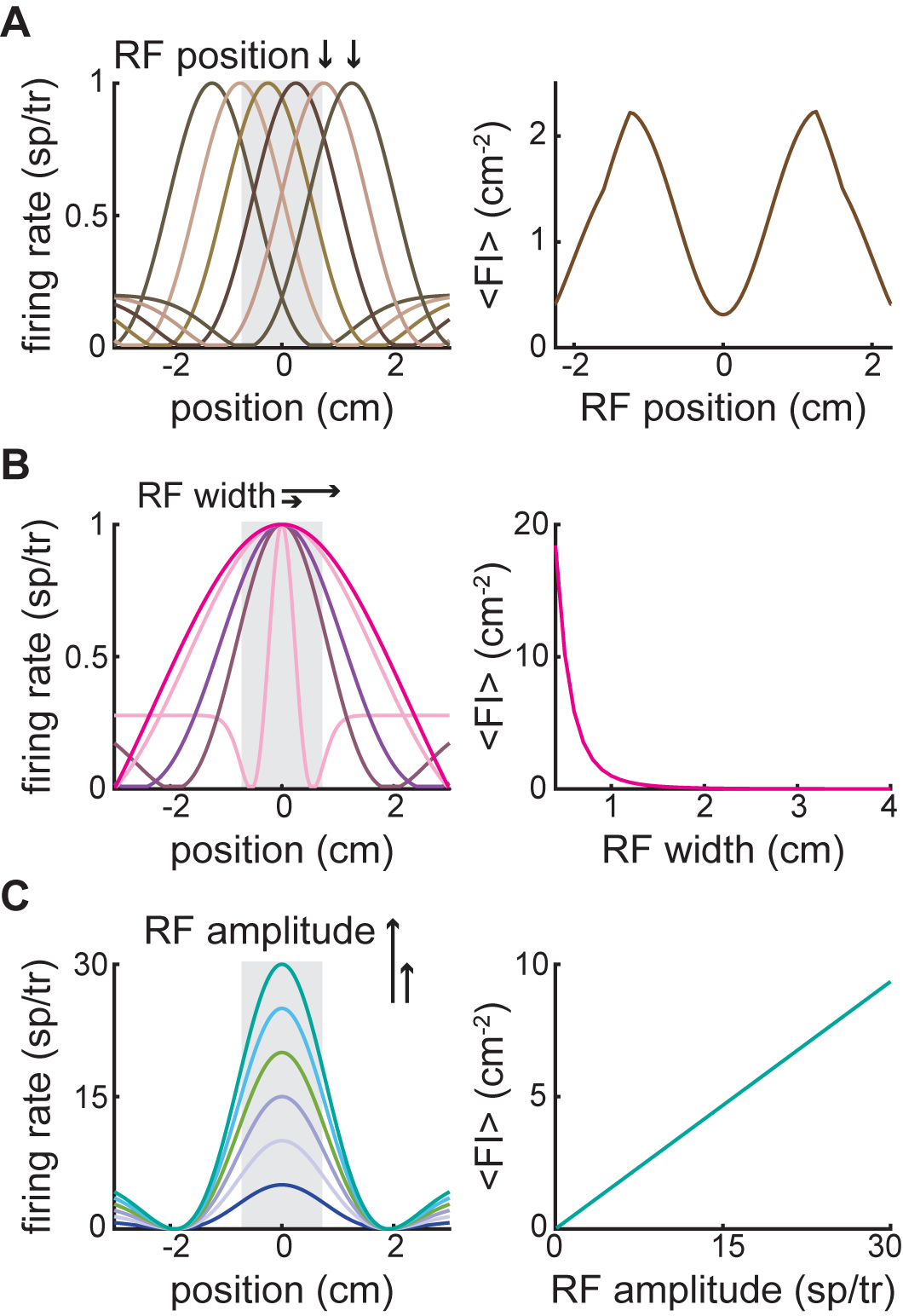

Supplement: S7 Fig — The left column shows example receptive fields, and the right column shows the Fisher information () averaged between -0.5 and 0.5 cm (grey region in left panels). (A) The receptive field position parameter is varied, while holding the receptive field width and amplitude parameters constant. (B) The receptive field width parameter is varied, while hold position and amplitude constant. (C) The receptive field amplitude parameter is varied while hold position and width constant. (TIF) [file pcbi.1010938.s007.tif]
