# Supplementary material for: Coding of object location by heterogeneous neural populations with spatially dependent correlations in weakly electric fish
Source: PLoS Comput Biol. 2023 Mar 3;19(3):e1010938. doi: 10.1371/journal.pcbi.1010938 (PMC10016687; doi:10.1371/journal.pcbi.1010938)
Supplement: Attachment — Submitted filename: Response_reviewer_FINALFINAL.docx [file pcbi.1010938.s008.docx]

**Reviewer #1:** The authors study the spatial dependence of correlations in the weakly electric fish, and the role of this structure in coding accuracy. They find that correlations reduce accuracy overall, but their spatial dependence increases accuracy as quantified by Fisher Information. They reproduce these effects in a computational model and explore the role of receptive field heterogeneity.

The problem is well motivated and the results are clearly presented. The authors use well known approaches to answer their question in a straightforward manner, which I view as a strength of the paper. The manuscript is suitable for publication in PLoS Computational Biology. I only have a few very minor suggestions that could help improve the paper.

>> We thank the reviewer for their encouraging comments.

1. Line 178: Specify what measure of covariance is used (e.g., spike count covariance) and point to the Methods.

>> We now mention that spike-count covariance is the measure of covariance and point to the Methods as requested (line 176).

2. Figure 5A: It would be interesting to also see the raw correlation values (without the absolute value), even if only as a Supplementary Figure. This could be important because negative correlations impact coding differently than positive correlations (see, e.g., Moreno-Bote et al, 2014). If the average sign of correlations differs based on whether neurons are ON-ON, ON-OFF, or OFF-OFF pairs, then these values could be shown as different colored points in the scatter plot.

>> We agree and now show the raw correlation values for same type (i.e., ON-ON and OFF-OFF) and opposite type (i.e., ON-OFF) pairs as different colored points in Fig. S2. We also mention that, while opposite-type pairs have negative correlations, it is important to note that their tuning curves are also opposite to one another (i.e., are negatively correlated). Therefore, the relationship between raw correlation values and tuning curves are similar for opposite and same-type pairs. This is now mentioned in the methods (lines 641-653) and in the figure caption of the Fig S2 (lines 1192-1200).

3. Figure 5A: Since the scatter plot is so noisy. it would be nice to see a curve fit to the scatter plot. This could also be done for the Supplementary Figure suggested above.

>> As suggested, we added a fitted curve to the scatter plots in Figure 5A as well as in Fig. S2. A description of the function used to fit the data has been added to the methods (lines 641-653).

**Reviewer #2:** Summary:
In the present study, Haggard and Chacron analyze the effects of correlations in the activity of neurons from the electric fish Apteronotus leptorhynchus in the coding of the spatial location of a stimulus along the rostro-caudal axis of the animal. The authors report that the neural activity presents spatially structures correlation patterns, and that these patterns allow to resolve the problem poised by information limiting correlations. Furthermore, through mathematical modeling the authors show that heterogeneity in the neural tuning and neural response magnitude help to optimize information transmission.
Strengths:
Overall, this is a well-executed study and a very nice manuscript. One of its main strengths is the use of a “non-standard” experimental model in the neutral coding literature. This allows to study the effects of receptive field heterogeneity in information transmission. While similar work has been done for other sensory systems in mammals, the use of the electro-sensory system in this model allows to control variables that are more difficult to manipulate in other systems/organisms.
Weaknesses:
The size of the studied neural populations is small, compared to the population sizes recorded in similar studies. Also, the manipulations performed to compute 1) independent and 2) spatially independent Fischer information are in a completely theoretical way. Although this is perfectly valid, it would be good to do a data shuffling instead of manually manipulating the covariance matrices. Similar results would be expected from both approaches.

>> We thank the reviewer for their encouraging comments. We have significantly modified the manuscript to address the weaknesses mentioned above. Specifically, we have shuffled the data as requested and have found similar results as expected (Methods: lines 719-722). Moreover, we now note that the population sizes are similar to those recorded in previous studies in this system (Methods: lines 625-626).

Major comments:
1) Line 228: The authors report that one of the main findings is that the spatial dependence of the covariance is due to the spatial dependency of noise correlations. Why is this surprising? Given that correlation is defined as the normalized covariance, it is only expected that if one of them shows spatial structure, the same will be observed in the other.

>> We understand the reviewer’s comment. We now emphasize in the text that, because the correlation coefficient is defined as the covariance-to-variance ratio, covariance and correlation can, in theory, have qualitatively different behaviors (lines 203-204). For example, if covariance and variance increase by a similar factor, then correlation would not change.

2) Lines 264-279: As mentioned above in “Weaknesses”. It is important to have a complimentary analysis in which independence and spatial independence are achieved through data shuffling, rather than direct manipulation of the covariance matrices. This way we would have “independent” information levels based on the data available, rather than the idealized scenario that results from setting off diagonal values to exactly zero by hand.

>> We agree with the reviewer and have compared results obtained when shuffling the data. As mentioned above, this led to similar results as expected (lines 719-722).

Minor comments:
3) Line 212: Please indicate what was the threshold here. I know it is in the methods, but there is no reason to bury that number in there.

>> We now state the threshold value in the text as requested (line 212).

4) Figure 5 legend (line 253) and Methods (line 790): please explain the criteria used to reject outliers (sig level, k)

>> We now mention how outliers were rejected in the methods (lines 846-849) and refer to the methods in the caption of Fig. 5.

5) Figure 6C (line 292): what was the population size for this figure?

>> We now mention the population size of 28 neurons in the legend (line 293).

6) Figure 8: How exactly was heterogeneity quantified? What does the horizontal axis in the plots indicate?

>> We have now clarified that heterogeneity is quantified by the standard deviation of the distribution from which the receptive field attribute (i.e., either of position, width, or amplitude) is drawn from in the results (lines 355-356, 388-389, and 404-405) and figure caption (lines 377-378).

7) Lines 480-482: It is a matter of debate if correlations have a role, or if they are simply due to the data processing inequality principle. There is certainly evidence that in some cases correlations are actively shaped to enhance specific information, but there are instances in which this is not clear. For the sake of completeness, it would be good to mention both sides.

>> We agree and have rewritten this section accordingly (lines 487-488).

8) Line 661: a clearer explanation of the procedure to calculate the slope of the receptive field is needed.

>> We have now clarified on the receptive field slope was computed (Lines 710-712).

9) Line 740: where do the slope and intercept for the linear model come from? For all other parameters it is stated that they are derived from the observed neural activity, one would assume the same applies for this linear model. Better to explicitly indicate the source.

>> We now clearly mention that parameter values were obtained by fitting a linear function to the data (lines 793-794).

10) Line 789: “normalcy” should be “normality”.

>> Thank you. This has been corrected (line 843).

**Reviewer #3:** This paper contains a masterful analysis of population coding of object location in the electrosensory system of the electric fish.

The conclusions about advantages for coding of heterogeneity of receptive fields and of spatially dependent correlations are well sustained by the data and are of wide interest.

The experiments and analyses are excellent and the paper is clear and well structured. The only suggestions for improvement (listed below) regard textual clarifications of specific aspects and better placing the paper in the context of the existing literature and better individuating its novelty.

>> We thank the reviewer for their positive comments which have helped improve our manuscript.

One main conclusion is about the advantages for population codes of heterogeneous neural properties. Advantages for heterogeneous properties have been shown before by theoretical studies. Some of these are cited by the authors (Shamir and Sompolinksy 2006). Some other prominent papers on heterogeneity and population coding are not cited, for example:

Ecker, A. S., Berens, P., Tolias, A. S. & Bethge, M. The effect of noise correlations in populations of diversely tuned neurons. J. Neurosci. 31, 14272–14283 (2011).

Wilke, S. D. & Eurich, C. W. Representational accuracy of stochastic neural populations. Neural Comput. 14, 155–189 (2002).

It would be useful to cite also these papers and to better discuss the specific new insights of the present paper with respect to the older theories of heterogeneity vs population codes.

>> We thank the reviewer for pointing this out. We now cite these papers and have modified the text to better explain the relationship between these and our current study in the discussion (lines 523-534).

The second main conclusion is about the location dependence of spike count correlations and its effect on decreasing the information-limiting effects of positive signal-noise correlations (“mitigating redundancy”). A location dependence of noise correlations which increases the population information has been reported (both among neurons, among astrocytes, and between neurons and astrocytes) in the hippocampus of mice during spatial navigation (Curreli et al PloS Biology 2022). More generally, beyond location coding, the problem of how modulations of correlations influence positively population codes has been the subject of much theoretical work, very relevant to the results presented here (e.g. Pola et al Network 2003, Josic, Shea- Brown, Doiron, de la Rocha,
Neural Comput. 2009, Azeredo da Silveira and Rieke Annu. Rev. Neurosci. 2021 and cited references). It would be interesting if the authors cite other evidence of information-enhancing location modulations of correlations and their contribution to coding (including the one suggested above) and discuss more differences, similarities, relationships and implications of their results with respect to previous works reporting related findings or theoretical considerations on stimulus-dependent correlations.

>> We agree with the reviewer and now discuss the differences and similarities between our study and previous work in detail in the discussion (lines 501-517).

On page 29 (Discussion), the authors speculate that noise correlations may be good for perceptual tasks even if they decrease information. Recent evidence to support this was presented in Valente et al Nature Neurosci 2021, which should be mentioned. Also, it should be mentioned that correlations may not only increase learning rate and robustness but also signal propagation (as shown the 1900s studies of Christof Koch, Emilio Salinas etc).

>> Thank you. We have modified the text accordingly (lines 491-493).

Introduction and abstract place a lot of emphasis on the role of population codes not only for information encoding but also for generating behaviors, which is a nice question. However, in my understanding, the paper results are more focused on information coding than in the subsequent generation of behaviors. It may be worth reconsidering the balance of the opening sentence of the abstract and introduction.

>> We have changed the abstract and introduction accordingly, and now limit the implications for behavior to the discussion.

The title is nice but al little too generic. I feel that having in the title of the paper object locations and electric fish would help readers.

>> We have changed the title to “Coding of object location by heterogeneous neural populations with spatially dependent correlations in weakly electric fish” accordingly.
